# Capgras delusion and auditory hallucinations in old age: a case of paraphrenia?

**DOI:** 10.1192/j.eurpsy.2023.1977

**Published:** 2023-07-19

**Authors:** J. P. N. Azenha, J. B. Rodrigues

**Affiliations:** Departamento de Psiquiatria, Centro Hospitalar de Lisboa Ocidental, Lisboa, Portugal

## Abstract

**Introduction:**

The term paraphrenia, as classically described by Kraepelin, characterizes a disorder that fits into the complex group of late onset psychoses and resembles schizophrenia, but with better preservation of affect and volition and less deterioration of personality. Over the last few decades, the concept has suffered several setbacks and is not currently recognised by the main manuals of mental disorders. However, there are several authors who argue that the diagnosis of paraphrenia has not lost its usefulness. In 1999, Munro and colleagues proposed a set of criteria to identify this entity and delimit it from the diagnoses of schizophrenia and delusional disorder.

**Objectives:**

Based on a clinical case, we intend to discuss the applicability of the criteria proposed by Munro and the usefulness of the concept of paraphrenia nowadays.

**Methods:**

Case report.

**Results:**

A 71-year-old woman was taken to the emergency department for presenting a first psychotic episode characterized by auditory hallucinations, persecutory delusional ideation and Capgras delirium. The delirium was well structured and very dynamic, interfering in the patient’s social and family spheres. Affects were preserved and adequate and no volitional alterations, thought forms or cognitive deficits were found. Organic pathology was also excluded. Thus, it was possible to make the diagnosis of paraphrenia in light of Munro’s criteria.

**Conclusions:**

The description of this case illustrates the definition and identification of paraphrenia, highlighting the usefulness of the proposed criteria and the importance of giving greater recognition to this entity in order to stimulate future research.

**Disclosure of Interest:**

None Declared

